# Import of proteins into the peroxisomal matrix

**DOI:** 10.3389/fphys.2013.00261

**Published:** 2013-09-24

**Authors:** Sohel Hasan, Harald W. Platta, Ralf Erdmann

**Affiliations:** ^1^Systembiochemie, Medizinische Fakultät, Ruhr-Universität BochumBochum, Germany; ^2^Biochemie Intrazellulärer Transportprozesse, Medizinische Fakultät, Ruhr-Universität BochumBochum, Germany

**Keywords:** peroxisome, protein import, ubiquitination, biogenesis, translocation, targeting

## Abstract

Peroxisomes constitute a dynamic compartment in all nucleated cells. They fulfill diverse metabolic tasks in response to environmental changes and cellular demands. This adaptation is implemented by modulation of the enzyme content of the organelles, which is accomplished by dynamically operating peroxisomal protein transport machineries. Soluble import receptors recognize their newly synthesized cargo proteins in the cytosol and ferry them to the peroxisomal membrane. Subsequently, the cargo is translocated into the matrix, where the receptor is ubiquitinated and exported back to the cytosol for further rounds of matrix protein import. This review discusses the recent progress in our understanding of the peroxisomal matrix protein import and its regulation by ubiquitination events as well as the current view on the translocation mechanism of folded proteins into peroxisomes. This article is part of a Special Issue entitled: Origin and spatiotemporal dynamics of the peroxisomal endomembrane system.

## Introduction

Peroxisomes are organelles that can be found in all nucleated cells. The number and morphology of peroxisomes varies significantly among different cell types, tissues or species. Peroxisomes are typically spherical organelles with a diameter from 0.1 to 1 μm that are surrounded by a single phospholipid bilayer membrane.

The peroxisomal luminal proteins are tightly packed in electron-dense, sometimes even crystalline matrix. The enzyme content varies depending on the cellular demands with the ability to adjust to the metabolic requirements of the cell. Accordingly, peroxisomes are considered to be multi-purpose organelles that contribute to the adaptation of cells to different environmental conditions. The peroxisomal matrix can harbor at least 50 different enzymes that are involved in diverse biochemical processes, such as beta-oxidation of fatty acids and the detoxification of hydrogen peroxide, which are considered as a main functions of peroxisomes (Schlüter et al., 2010). The beta-oxidation of fatty acids exclusively takes place in peroxisomes in yeast and plants, while in the case of mammalian cells, only the very long chain fatty acids (VLCF) are oxidized in peroxisomes, whereas shorter chain fatty acids are oxidized in mitochondria. Additionally, it has been established that peroxisomes are required for the synthesis of plasmalogens and bile acids in mammals (Wanders and Waterham, 2006) and that they contribute to certain biochemical steps of photorespiration of plants (Hu et al., 2012) as well as the final steps of penicillin biosynthesis in some filamentous fungi (Meijer et al., 2010). More recently explored functions of peroxisomes include an partial involvement in Vitamin K biosynthesis in plants (Babujee et al., 2010; Widhalm et al., 2012), calcium homeostasis in mammals (Lasorsa et al., 2008) as well as pheromone production in nematodes and insects (Joo et al., 2010; Spiegel et al., 2011). Moreover, peroxisomes contribute to the iron uptake and therefore virulence of pathogenic *Aspergillus* species, by containing enzymes required for the biosynthesis of siderophores (Gründlinger et al., 2013).

Typical peroxisomes together with specialized peroxisomes like glyoxysomes, glycosomes and Woronin bodies constitute the organelle family of “microbodies,” whose members are all evolutionary related, sometimes even interconvertible compartments. The glycosomes of the protist order Kinetoplastida harbor glycolysis enzymes, whereas the glyoxysomes of the germinating plant seeds house enzymes of the glyoxylate cycle, and the Woronin bodies of filamentous fungi are tightly packed with a crystalline protein and function to seal septal pores in response to wounding (Pieuchot and Jedd, 2012).

The pivotal role of the correct topogenesis of peroxisomal proteins is also pointed out by the fact that dysfunction of human peroxisomes is associated with a spectrum of severe peroxisomal disorders, like e.g., Zellweger syndrome or X-linked adrenoleukodystrophy (Baes and Van Veldhoven, 2012; Nagotu et al., 2012; Poll-the and Gärtner, 2012; Waterham and Ebberink, 2012). These different disorders have in common that they are characterized by an abnormal peroxisome assembly and impaired peroxisomal function, in many cases resulting in multisystemic disorders that lead to death in early infancy. Furthermore, recent data demonstrate a link of peroxisome function and antiviral innate immunity as that they can promote a rapid response to viral infection via peroxisomal antiviral signaling proteins (Dixit et al., 2010). In addition, it has been demonstrated that functional peroxisomes counteract the progressive brain damage and cognitive decline found in Alzheimer's disease (Kou et al., 2011; Lizard et al., 2012). Finally, the reactive oxygene metabolism connects peroxisomes to the molecular process of aging (Giordano and Terlecky, 2012; Manivannan et al., 2012).

All of the mentioned physiologic tasks strictly depend on a proper compartmentalization of the corresponding enzymes, which itself relies on a proper peroxisomal biogenesis.

The biogenesis of peroxisomes conceptually consists of the (1) formation and proliferation of the peroxisomal membrane, (2) peroxisome movement and inheritance as well as (3) the topogenesis of peroxisomal membrane and (4) matrix proteins (Platta and Erdmann, 2007; Fagarasanu et al., 2010; Islinger et al., 2012; Liu et al., 2012; Theodoulou et al., 2013). These tasks are essentially carried out by peroxisomal biogenesis factors, the peroxins. From the 34 peroxins described so far, at least 19 are known to be directly involved in different stages of peroxisomal matrix protein import (Table 1). In this review, we will focus on the recent developments concerning the topogenesis of peroxisomal matrix proteins.

**Table 1:** **Peroxins involved in peroxisomal matrix protein import**.

**Peroxin**	**Enzyme/activity**	**Mw (KDa)**	**Function**
Pex1p (PAS1)	AAA-type ATPase	117.3	Binds Pex6p and is involved in the dislocation of the PTS-receptors
Pex2p (CRT1, PAS5)	Ubiquitin-protein ligase (E3), RING-domain	30.8	Forms together with Pex10p and Pex12p the RING-complex and is involved in the ubiquitination of the PTS-receptors
Pex4p (PAS2, UBC10)	Ubiquitin-conjugating enzyme (E2)	21.1	Monoubiquitination of the PTS-receptors
Pex5p (PAS10)	PTS1 receptor, TPR-domain	69.3	Receptor for the PTS1-signal; required for the PTS1-dependent matrix protein import
Pex6p (PAS8)	AAA-type ATPase	115.6	Binds Pex1p and is involved in the dislocation of the PTS-receptors
Pex7p (PAS7, PEB1)	PTS2 receptor, WD40 domain	42.3	Receptor for the PTS2-signal; required for the PTS2-dependent matrix protein import; forms complex with PTS2-co-receptors (Pex18p/Pex20p/Pex21p)
Pex8p (PAS6)	Coordinator of protein import machinery	68.2	Peripheral intraperoxisomal membrane protein; bridges docking- and RING-complex; possibly involved in cargo disassembly
Pex10p (PAS4)	Ubiquitin-protein ligase (E3), RING-domain	39.1	Forms together with Pex2p and Pex12p the RING-complex and is involved in the ubiquitination of the PTS-receptors
Pex12p (PAS11)	Ubiquitin-protein ligase (E3), RING-domain	46.0	Forms together with Pex2p and Pex10p the RING-complex and is involved in the ubiquitination of the PTS-receptors
Pex13p (PAS20)	Core component of docking machinery, SH3-domain	42.7	Integral membrane protein required for the docking of receptor/cargo complexes; forms docking complex with Pex14p and Pex17p
Pex14p	Core component of docking machinery, PXXP-domain	43.7	Membrane protein required for the docking of receptor/cargo complexes; forms docking complex with Pex13p and Pex17p
Pex15p (PAS21)	Membrane anchor of the AAA-peroxins	43.7	Phosphorylated tail-anchored PMP that is involve in the recruitment of yeast Pex6p to the peroxisomal membrane
Pex17p (PAS9)	Docking complex	23.2	Membrane-associated protein that forms the docking complex with Pex14p and Pex17p
Pex18p	PTS2-co-receptor	32.0	Interacts with PTS2-receptor Pex7p; partially redundant with Pex21p (in *S. cerevisiae*)
Pex20p	PTS2-co-receptor	37.8	Involved in PTS2-dependent protein import, mostly as co-receptor of Pex7p (in most fungi, like e.g., *P. pastoris*)
Pex21p	PTS2 protein import	33.0	Interacts with PTS2-receptor Pex7p; partially redundant with Pex21p (in *S. cerevisiae*)
Pex22p (YAF5)	Peroxisomal protein import	19.9	Membrane protein required the recruitment of Pex4p to the peroxisomal membrane
Pex26p	Membrane anchor of the AAA-peroxins	33.9	Tail-anchored PMP that is involved in the recruitment of mammalian Pex6p to peroxisomal membrane
Pex33p (Pex14/17p)	Docking complex	52.5	Part of the docking complex in filamentous fungi, e.g., *N. crassa*

The names in brackets refer to the previous original designations in yeast. Except where indicated, the molecular mass refers to the S. cerevisiae protein.

## Matrix protein import

Because peroxisomes do not contain genetic material, all of their protein content is encoded in the nucleus, synthesized on free polyribosomes in the cytosol and targeted to the organelle in a post-translational manner [reviewed in Leon et al. (2006a)]. Recent data suggest that already the mRNA of most of the analyzed peroxisomal proteins is targeted to the proximity area of the peroxisome prior to the translation step (Zipor et al., 2009, 2011). The capacity to import proteins in a fully folded or even oligomeric and co-factor bound state is an extraordinary feature of peroxisomes and differentiates peroxisomes from other classical organelles like mitochondria or chloroplasts [reviewed in Leon et al. (2006a)]. While this concept had been established quite early, the question whether folded cargo proteins are preferably imported in an oligomeric or monomeric state has been picked up by two recent studies. While one study finds that mammalian Pex5p strictly imports tetrameric catalase (Otera and Fujiki, 2011), another study suggests that the catalase oligomer may be disassembled during the import process (Freitas et al., 2011).

**Figure 1:**
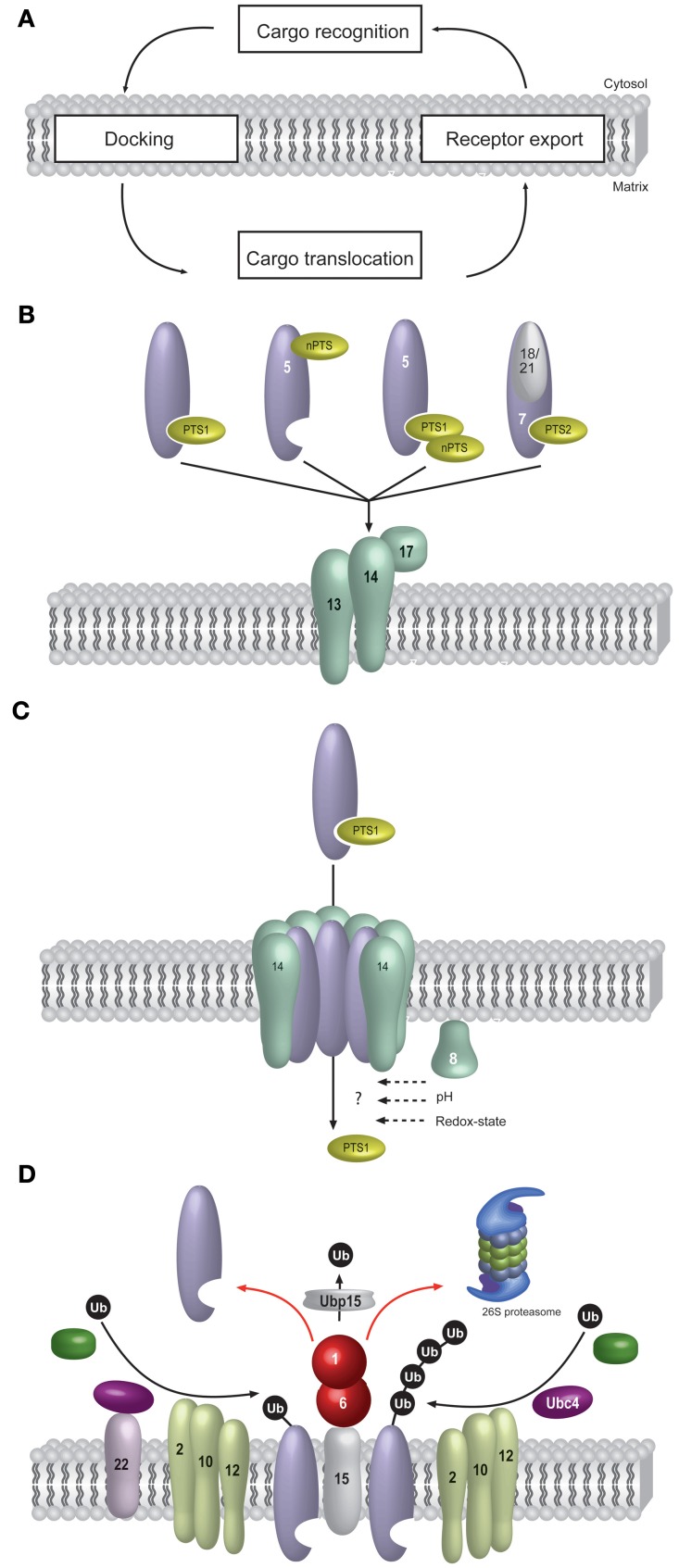
**Matrix protein import into peroxisomes.** Most principles of peroxisomal protein import are evolutionary conserved. The schematic representation shown here refers to the situation in *Saccharomyces cerevisiae*. Peroxisomal matrix protein import takes place posttranslationally and requires an elaborate protein import machinery, consisting of peroxisome biogenesis factors, so called peroxins. **(A)** Another feature of peroxisomal import is the requirement for cycling receptors. The import process can conceptually be divided into cargo-recognition by the receptors in the cytosol, docking of the receptor/cargo-complex at the peroxisomal membrane, cargo translocation and finally export of the receptor back to the cytosol. **(B)** Cargo-recognition and docking: Proteins harboring a peroxisomal targeting signal of type 1 (PTS1) or type II (PTS2) are recognized in the cytosol by specific import receptors, Pex5p and Pex7p, respectively. Alternatively, some cargo proteins do not harbor a PTS or do not essential depend on it. Some of these non-PTS proteins (nPTS) bind to the N-terminus of Pex5p or to canonical PTS1-proteins. The cargo-loaded receptors are directed to a docking complex at the peroxisomal membrane. For this, the PTS2-receptor Pex7p requires auxiliary proteins, in baker's yeast these are the redundant Pex18p or Pex21p. The receptor-cargo-complexes bind to the docking-complex (Pex13p, Pex14p and Pex17p) at the peroxisomal membrane. The following steps are better known for the PTS1- than the PTS2-pathway. **(C)** Cargo-translocation: It is assumed that the association of Pex14p and cargo-loaded Pex5p leads to the formation of a transient pore, which functions as a protein-conducting channel. The cargo is translocated into the peroxisomal lumen in an unknown manner. In intraperoxisomal Pex8p, a pH-shift or the redox-state might be involved in receptor-cargo dissociation. **(D)** At the end of the import cascade, the receptor is recycled from the peroxisomal membrane back to the cytosol for another round of import. For this, Pex5p is monoubiquitinated by the Pex22p-anchored ubiquitin-conjugating enzyme Pex4p and the ubiquitin-protein ligase Pex12p, which forms the RING-finger complex together with the other ubiquitin-protein ligases Pex2p and Pex10p. Pex5p-polyubiquitination is performed by the ubiquitin-conjugating enzyme Ubc4p in conjunction with the ubiquitin-protein ligases Pex2p and Pex10p. For Pex5p, it has been demonstrated that the ubiquitin-signal leads to an ATP-dependent dislocation of Pex5p from the peroxisomal membrane. This process is performed by the AAA-type ATPases Pex1p and Pex6p, which are anchored to the peroxisomal membrane via Pex15p. During or shortly after receptor export to the cytosol, the ubiquitin is removed by the ubiquitin-hydrolase Ubp15p.

In general, peroxisomes share the capability to translocate folded proteins with the Twin-Arg-Translocation (Tat) pathway of bacteria and thylakoid membranes (Albiniak et al., 2012; Palmer and Berks, 2012). However, while the entire machinery required for the translocation of the substrate protein is membrane-bound in the case of the Tat-system, some of the constituents of the peroxisomal translocation apparatus are soluble (Schnell and Hebert, 2003). The peroxisomal import receptors cycle between the cytosol and the peroxisomal membrane (Dammai and Subramani, 2001; Nair et al., 2004). According to the dynamics of the import receptors, the protein import process into peroxisomes can be divided into five stages such as (1) cargo recognition in the cytosol, (2) docking of the receptor/cargo- complex at the peroxisomal membrane, (3) cargo translocation over the membrane, (4) release of the cargo into the peroxisomal matrix, and (5) receptor recycling (Figure 1A).

### Cargo recognition in the cytosol

Newly synthesized peroxisomal matrix proteins are transported to their destination by means of a targeting sequence. The majority of the matrix proteins harbors a peroxisomal targeting signal type 1 (**PTS1**) at the carboxy-terminus, which is defined by the amino acids SKL and variants of the motif fitting the consensus (S/A/C)-(K/R/H)-(L/A). Because also additional adjacent residues have an impact on the cargo-recognition, the definition of the PTS1 can be extended to a dodecamer (Brocard and Hartig, 2006; Chowdhary et al., 2012). The conserved receptor for the PTS1-signal is **Pex5p**, which recognizes the PTS1-sequence via a tetratricopeptide repeats (TPRs) containing domain within its carboxy-terminal half. Crystal structures of the cargo-loaded and unloaded PTS1-receptor revealed that cargo binding induces major conformational changes within the receptor, which might generate a docking-competent state of the receptor (Stanley et al., 2006; Shiozawa et al., 2009; Fodor et al., 2012).

The peroxisomal targeting signal type 2 (**PTS2**) sequence is the second known peroxisomal targeting determinant. It is usually located within the first 20 amino acids of the cargo protein and has been defined as a nona-peptide by the amino acid signal (RK)-(LVIQ)-XX-(LVIHQ)-(LSGAK)-X-(HQ)-(LAF) (Petriv et al., 2004; Lazarow, 2006). In plants, approximately one third of peroxisomal matrix proteins harbor a PTS2-signal (Lingner et al., 2011; Chowdhary et al., 2012), whereas in *Saccharomyces cerevisiae* only three proteins are known to use this targeting sequence (Grunau et al., 2009; Jung et al., 2010). The PTS2-pathway is completely absent in *Caenorhabditis elegans* (Motley et al., 2000), *Drosophila melanogaster* (Faust et al., 2012) and the protist *Phaeodactylum tricornutum* (Gonzalez et al., 2011), which therefore import all matrix proteins via the PTS1-pathway.

The PTS2-cargo is recognized by **Pex7p**, which contains several tryptophan-aspartic acid (WD) repeats that mediate the binding. However, unlike the PTS1-receptor, Pex7p is necessary, but not sufficient to carry out all steps of the import process because it requires auxiliary proteins. These PTS2-co-receptors are the redundant **Pex18p** and **Pex21p** in *S. cerevisiae*, the orthologous **Pex20p** in most other yeasts and fungi as well as **Pex5L**, the longer of two splice isoforms of Pex5p, in mammals and plants (Schliebs and Kunau, 2006). Interestingly, *Podospora anserina* Pex20p has been reported to carry out a Pex7p-dependent function in matrix protein import as well as a Pex7p-independent function in meiocyte formation (Peraza-Reyes et al., 2011).

The targeting of a subset of peroxisomal matrix proteins does not essentially rely on one of the two classical targeting signals (Figure 1B). Some of these proteins can be co-imported via an association with canonical PTS-cargo proteins. This “piggy-back import” has been demonstrated for the enoyl-CoA isomerases Eci1p and Dci1p from *S. cerevisiae* (Yang et al., 2001) and the five acyl-CoA oxidase isoforms from *Yarrowia lipolytica* (Titorenko et al., 2002) and more recently also for mammalian Cu/Zn superoxide dismutase (Islinger et al., 2009). Other proteins, like acyl-CoA oxidase from *S. cerevisiae* or alcohol oxidase from *H. polymorpha* (Klein et al., 2002; Gunkel et al., 2004), interact directly with Pex5p in a PTS1-independent fashion via the binding to the N-terminal region of Pex5p in a process, which is called “**non-PTS import**” (Van Der Klei and Veenhuis, 2006).

A new chapter of peroxisomal targeting signals has recently been opened by the finding that glycolytic enzymes of the analyzed fungi and mammalian species contain a cryptic PTS (Freitag et al., 2012). With exception of the well-established glycolytic enzymes found in glycosomes of Kinetoplastids (Gualdrón-López et al., 2012) and the report on the peroxisome-dependent glucose metabolism of the fungus *Cryptococcus neoformans* (Idnurm et al., 2007), these enzyme were thought to be strictly cytosolic in all species. However, they contain a cryptic peroxisomal targeting signal, which can be generated or eliminated in a species-specific manner by ribosomal read-through or alternative splicing (Freitag et al., 2012). This differential targeting to two locations may indicate a dynamic regulation of glycolysis by sequestering the key enzymes away from the cytosol.

### Association of the cargo-loaded receptor with the membrane via the docking complex

Several lines of evidence indicate that only the cargo-bound receptors are efficiently directed to the peroxisome (Gouveia et al., 2003; Grunau et al., 2009). The cargo-bound receptors associate with the peroxisomal membrane via a docking complex (Figure 1B), which consists in all known species of the core components Pex13p and Pex14p (Kiel et al., 2006). The absence of either Pex13p or Pex14p significantly affects the import pathway of cargos targeted to the peroxisome (Azevedo and Schliebs, 2006; Williams and Distel, 2006). **Pex13p** is an integral membrane protein, which binds to Pex14p via its SH3-domain and also via an intraperoxisomal binding site (Pires et al., 2003; Schell-Steven et al., 2005). The N-terminal part of Pex13p also binds to the PTS2-receptor, while the SH3-domain of the yeast protein contains a binding site for the PTS1 receptor Pex5p (Williams and Distel, 2006). **Pex14p** contains a proline-rich segment for binding of the SH3-domain of Pex13p. Pex14p has been described as an carbonate-resistant integral membrane protein but in some species, it behaves like a peripheral protein (Azevedo and Schliebs, 2006). As Pex13p also Pex14p binds both PTS-receptors at different sites (Niederhoff et al., 2005). Nuclear magnetic resonance (NMR) and determination of the crystal structures (Neufeld et al., 2009; Su et al., 2009) revealed that the Pex5p/Pex14p interface comprises two hydrophobic cavities of Pex14p, which bind characteristic WXXXF/Y motifs of PTS1 receptor Pex5p. In general, Pex14p is considered to be the initial binding partner for the cargo-bound PTS1-receptor. However, because the available data addressing this question are limited and because of the observation that Pex13p can also associate with cargo-bound PTS-receptors (Grunau et al., 2009; Natsuyama et al., 2013), the individual contribution of Pex13p and Pex14p to the initial docking event remains to be further investigated.

In many species, the docking complex contains further peroxins in addition to Pex13p and Pex14p. Yeast **Pex17p** is a peripheral membrane protein of unknown function, which associates with peroxisomes via Pex14p but does not interact with Pex5p. A deficiency in Pex17p affects import of PTS1 as well as PTS2 proteins by an unknown mechanism (Azevedo and Schliebs, 2006). A homolog of Pex17p in higher eukaryotes has not yet been identified (Kiel et al., 2006). However, in filamentous fungi, a chimeric protein that consists of a Pex14p-like amino-terminal domain and a Pex17p-like carboxyl-terminal domain has been described (Managadze et al., 2010; Opaliński et al., 2010; Peraza-Reyes et al., 2011). This chimeric protein is called Pex14/17p in *P. anserina* and *Penicilium crysogenum*, while it is named Pex33p in *Neurospora crassa*.

*Trypanosoma brucei* contains two very different isoforms of Pex13p. While PEX13.1 resembles the conserved Pex13p, the PEX13.2 lacks the SH3 domain and contains a PTS1-signal at its carboxyl-terminus (Brennand et al., 2012; Verplaetse et al., 2012). It will be of interest to elucidate the special contribution of PEX13.2 to the protein import process, especially as it is part of the docking complex and essential for glycosome biogenesis.

A recent study of a Zellweger spectrum patient cell line describes the dimerization of human Pex13p (Krause et al., 2013). This dimerization occurs independently of the interaction to Pex14p and is required for PTS1-protein import (Krause et al., 2013). Also Pex14p can undergo dimerization, even though the functional impact is not understood yet (Su et al., 2010).

The association with the docking complex marks the entry of Pex5p to the protein-protein interaction network at the peroxisome (Hazra et al., 2002; Agne et al., 2003; Oeljeklaus et al., 2012). However, the collected data strongly indicate that components of the docking complex are far more than static receptor anchors and that they display a certain dynamic contribution to the peroxisomal protein import cascade.

### Translocation over the transient import pore and cargo-release into the matrix

The actual mode of matrix protein import is still elusive and how the cargo proteins traverse the peroxisomal membrane without affecting the permeability barrier remains hypothetical.

#### Transient import pore

Over the years, several models have been put forward of how folded and oligomeric proteins may traverse the peroxisomal membrane. The translocon has not yet been visualized and these models range from a channel consisting of multi-membrane spanning proteins and a transiently opened import pore to a pinocytosis-related model that completely lacks a translocon [as discussed in Girzalsky et al. (2009)].

The results collected in recent years strongly favor the idea of a transiently opened import pore (Figure 1C). It has been suggested that the major constituents of this dynamic pore may be membrane proteins Pex14p and Pex13p (Grou et al., 2009a) or Pex14p and the PTS1-receptor Pex5p (Erdmann and Schliebs, 2005). Indeed, one of the surprising features of Pex5p is that it can bind to lipids and change its topology at the peroxisomal membrane where it is partially carbonate resistant, adjusts to a partial protease-protected state and thereby behaves like an integral membrane protein (Gouveia et al., 2000, 2002; Platta et al., 2005; Kerssen et al., 2006). Furthermore, Pex5p together with Pex14p constitute the minimal unit for the translocation of the intraperoxisomal Pex8p across the membrane in *P. pastoris* (Ma et al., 2009). The functional interplay is further pointed out by the finding that at least in CHO cell lines, Pex5p can stabilize Pex14p (Natsuyama et al., 2013). *Leishmania donovani* Pex14p forms a homo-oligomeric complex, which undergoes major conformational changes upon Pex5p-binding (Cyr et al., 2008). Importantly, the Pex5p-Pex14p sub-complex of *S. cerevisiae* harbors pore forming activity in electrophysiological studies (Meinecke et al., 2010). This complex, which almost exclusively consists of Pex5p and Pex14p can be reconstituted into membranes and displays channel activity when incubated with a cargo-loaded soluble Pex5p (Meinecke et al., 2010). The size of this pore formed by the transiently gated ion conducting channel is variable up to 9 nm and therefore appears to accomplish the standards for the passage of folded proteins into the peroxisomal matrix. However, the exact composition of the pore as well as the driving force and the mechanism of cargo translocation remain elusive.

#### Cargo release

Data on how the cargo is released into the peroxisomal lumen are still scarce (Figure 1C). The first study to tackle this question directly suggests that the intraperoxisomal peripheral membrane protein **Pex8p** of *H. polymorpha* might be in involved in this process, because it can dissociate receptor-cargo complexes *in vitro* (Wang et al., 2003). In addition, the same study suggested that a change in pH might induce a disassembly of Pex5p-oligomers into the monomeric form and thereby also induces the dissociation of the cargo from this complex (Wang et al., 2003). A recent study in *P. pastoris* suggests that Pex8p is involved in a redox-state dependent disassembly of Pex5p oligomers and cargo (Ma et al., 2013). However, it is not easy to generalize these results because the intraperoxisomal pH may vary significantly depending on the experimental condition (Visser et al., 2007) and because Pex8p is a less conserved yeast protein that seems to be absent in most other species (Kiel et al., 2006). *S. cerevisiae* Pex8p has been described to function as a structural link of the docking complex to the export machinery (Agne et al., 2003; Platta et al., 2013), while this task is accomplished by Pex3p in *P. pastoris* (Hazra et al., 2002). One hypothetical explanation could be that the proposed role of Pex8p in cargo release may be transferred to the conserved parts of the docking complex in other species. In this respect, it is interesting to note that a recent publication demonstrates that the amino-terminus of mammalian Pex14p plays a role in the release of Pex5p-bound PTS1-cargo from the translocation machinery into the peroxisomal matrix (Freitas et al., 2011).

The signal sequence of a subset of the imported proteins is proteolytically removed after the import in peroxisomes of mammals and plants (Kurochkin et al., 2007; Schuhmann et al., 2008; Okumoto et al., 2011a). In the case of mammalian beta-oxidation enzymes, the intraperoxisomal protease Tysnd1 is responsible both for the removal of the leader peptide from PTS2 proteins as well as for the processing of PTS1 proteins and controls thereby the proper activity of these enzymes in beta-oxidation (Okumoto et al., 2011a). Indeed, recent work demonstrates that a deficiency in Tysnd1 results in a mild Zellweger syndrome spectrum-resembling phenotype in mice (Mizuno et al., 2013).

### Ubiquitination and dislocation of the receptor by the exportomer

Subsequent to cargo release, the receptor is exported to the cytosol by a molecular machinery called the peroxisomal exportomer (Platta et al., 2013). This machinery comprises sub-complexes consisting of mechano-enzymes that provide the pulling-force to extract the receptor from the membrane as well as sub-complexes that are required for the generation of the export-signal, which is the ubiquitination of the receptor (Figure 1D).

#### Recycling pathway of the PTS1-receptor

Most work has been dedicated to the ubiquitination of the PTS1-receptor Pex5p. Under wild-type conditions, the major modification of Pex5p is the attachment of a single ubiquitin moiety on a conserved cysteine residue (Kragt et al., 2005; Carvalho et al., 2007; Williams et al., 2007; Okumoto et al., 2011b). This monoubiquitination represents a prerequisite for the export of Pex5p back to the cytosol, which represents a molecular requirement that seems to be conserved from yeast to man (Carvalho et al., 2007; Platta et al., 2007, 2008; Okumoto et al., 2011b). The ubiquitin-conjugating enzyme (E2) **Pex4p** together with its membrane anchor **Pex22p** are required for this modification in *S. cerevisiae* (Platta et al., 2007; Williams et al., 2007, 2012). Mammalian cells lack clear Pex4p- and Pex22p-orthologs and here the functional-related isoforms UbcH5a, UbcH5b and UbcH5c catalyze the cysteine-dependent monoubiquitination (Grou et al., 2008). Proper monoubiquitination of Pex5p depends on an intact RING-peroxin complex (Kragt et al., 2005; Williams et al., 2008; Platta et al., 2009). Work in *S. cerevisiae* has demonstrated that **Pex2p** and **Pex12p** (Platta et al., 2009) as well as **Pex10p** (Williams et al., 2008; Platta et al., 2009) display ubiquitin-protein ligase (E3) activity. Recently, it could be confirmed that also each of the *A. thaliana* RING-peroxins has E3 activity (Kaur et al., 2013). The deletion of one of the RING-peroxin genes causes the instability of the entire complex (Hazra et al., 2002; Agne et al., 2003) and therefore inhibition of monoubiquitination of Pex5p (Williams et al., 2008; Platta et al., 2009). However, *in vitro* ubiquitination studies with recombinant proteins as well as additional work with RING-peroxin truncations lacking the catalytic RING-domain suggest that Pex12p is the E3 ligase directly responsible for monoubiquitination of Pex5p (Platta et al., 2009). At least *in vitro*, the activity of Pex12p can be synergistically enhanced in presence of the RING-domain of Pex10p (El Magraoui et al., 2012).

Following its monoubiquitination, the PTS1-receptor is exported back to cytosol in an AAA (ATPases associated with diverse cellular activities)-complex dependent manner. The peroxisomal AAA-type ATPases **Pex1p** and **Pex6p** play a non-redundant role in this process and are supposed to act as dislocases that extract the Pex5p from the membrane (Miyata and Fujiki, 2005; Platta et al., 2005). They are anchored to the peroxisomal membrane by the tail-anchored protein **Pex15p** in yeast as well as the orthologous Pex26p in mammals and APEM9 in plants (Birschmann et al., 2003; Matsumoto et al., 2003; Goto et al., 2011; Nashiro et al., 2011). The binding and hydrolysis of ATP by Pex1p and Pex6p is supposed to induce conformational changes that generate the force for the pull the receptor out of the membrane (Fujiki et al., 2012; Grimm et al., 2012). Although monoubiquitination of the PTS1-receptor plays a crucial role in its release, the exact molecular mechanism of substrate recognition and extraction from the membrane is still unclear. Interestingly, recent work from mammalian cells identified AWP1, which binds both Pex6p as well as ubiquitin (Miyata et al., 2012). This finding strongly suggest that AWP1 might function as specific linker, which enables the AAA-peroxins to transfer their pulling force to the monoubiquitinated receptor, thereby driving its export.

#### Degradation pathway of the PTS1-receptor

The cysteine-dependent monoubiquitination of Pex5p primes the PTS1-receptor for its recycling under normal conditions. However, in cases where the recycling pathway is hampered, Pex5p is polyubiquitinated on lysine residues and finally degraded in the 26S proteasome (Platta et al., 2004, 2007; Kiel et al., 2005; Williams et al., 2007) (Figure 1D). The polyubiquitination of *S. cerevisiae* Pex5p mainly depends on the ubiquitin-conjugating enzyme (E2) **Ubc4p** and the partially redundant Ubc5p and Ubc1p (Platta et al., 2004; Kiel et al., 2005; Kragt et al., 2005). The RING-peroxins Pex10p (Williams et al., 2008) as well as Pex2p (Platta et al., 2009) have been suggested to function as corresponding ubiquitin-protein ligases (E3). It is interesting to note that a recent *in vitro* study demonstrates that Pex10p(RING) can enhance the ubiquitination activity of the Ubc4p-Pex2p(RING) unit (El Magraoui et al., 2012), indicating that possibly these two RING-peroxins act together in the catalysis of the Ubc4p-dependent ubiquitination. The proteasomal degradation of the polyubiquitinated *S. cerevisiae* Pex5p is supposed to represent a quality control system for aberrant PTS1-receptor molecules (Platta et al., 2013).

#### Deubiquitination

Pex5p found in the cytosol is not further ubiquitinated *in vivo*, indicating that the ubiquitin moiety is removed upon or shortly after membrane release of the receptor (Figure 1D). In general, ubiquitin hydrolases perform the cleavage of ubiquitin from substrates (Amerik and Hochstrasser, 2004). The corresponding deubiquitinating enzyme in *S. cerevisiae* for the removal of the ubiquitin moiety from the modified PTS1-receptor is **Ubp15p** (Debelyy et al., 2011), while the non-orthologous **USP9X** has been found to deubiquitinate the monoubiquitinated Pex5p in mammals (Grou et al., 2012). However, both studies suggest that also other deubiquitinating enzymes may act in a redundant manner on Pex5p. Furthermore, at least a minor fraction of the monoubiquitinated Pex5p can be deubiquitinated in a non-enzymatic manner by cleavage of the thioester bond between Pex5p and ubiquitin by a nucleophilic attack of glutathione (Grou et al., 2009b).

#### Functional role of ubiquitination in the PTS2-import pathway

The PTS2-co-receptor Pex18p of *S. cerevisiae* was the first peroxin that has been found to be ubiquitinated (Purdue and Lazarow, 2001). Recent studies analyzed the modified forms of PTS2-co-receptors in more detail. This resulted in the discovery that Pex18p as well as the orthologous *P. pastoris* Pex20p are ubiquitinated in a similar manner to the PTS1-receptor Pex5p. Both Pex18p and Pex20p are polyubiquitinated on lysine residues and monoubiquitinated on a cysteine. The monoubiquitination is essential for matrix protein import, while the polyubiquitination regulates the stability of the PTS2-co-receptors (Leon et al., 2006b; Hensel et al., 2011; Liu and Subramani, 2013). Pex4p, the E2 ubiquitin-conjugation enzyme and the RING peroxins (Pex2p, Pex10p, Pex12p) proved to be required for mono- and polyubiquitination of *P. pastoris* Pex20p (Liu and Subramani, 2013). It is interesting to note that Pex18p displays a constitutive turn-over even in wild-type cells, while the PTS2-receptor Pex7p is stable (Hensel et al., 2011). So far, no evidence for ubiquitination or degradation of Pex7p has been found in yeast. However, a recent report demonstrates that endogenous *Arabidopsis* Pex7p is degraded by the proteasome when the dominant-negative GFP-Pex7p is expressed and that this mechanism depends on the interaction of Pex7p with the Rab GTPase RabE1c (Cui et al., 2013). However, it remains to be elucidated whether the observed disposal of Pex7p is also present under normal conditions and if this pathway is conserved among species.

## Interconnection of receptor export and cargo import

The peroxisomal export machinery components and the mechanism by which they facilitate the cycling of peroxisomal receptors are evolutionary and functionally related to endoplasmic reticulum associated degradation (ERAD) (Schlüter et al., 2006; Gabaldón, 2010; Schliebs et al., 2010). ERAD signifies a mechanism by which accumulated misfolded and polyubiquitinated proteins are extracted from the ER membrane or lumen for their subsequent degradation by the proteasome (Hampton and Sommer, 2012). Both the peroxisomal protein import machinery as well as the ERAD machineries utilize ubiquitination to mark proteins for their ATP-dependent release from the membrane by AAA-type ATPases.

The collected evidence over the last years defined the peroxisomal exportomer as the energy-consuming entity for peroxisomal protein import. In general, this might indicate that the step of cargo translocation into the matrix itself is an ATP-dependent step. However, a study with a rat liver *in vitro* system suggests that cargo release may occur independently of ATP and therefore presumably before the ubiquitination step (Alencastre et al., 2009). A CHO cell *in vitro* system instead supports the model that cargo translocation relies on the hydrolysis of ATP (Miyata et al., 2009). Along this line, it has been suggested that the ERAD-like ATP-driven export of the ubiquitinated receptor might be mechanistically linked to cargo translocation, which is highlighted by the so called export-driven-import model (Schliebs et al., 2010). In support of this model, the presence of a functional receptor–export complex is a pre-requisite for the ATP-dependent import of matrix proteins into peroxisomes (Schliebs et al., 2010). Current work on the ubiquitination of the PTS2-co-receptor Pex18p in *S. cerevisiae* provided direct evidence for the export-driven-import model. The cysteine-dependent monoubiquitination of Pex18p, which is required for the export of the co-receptor from the peroxisomal membrane into the cytosol, was observed to be a prerequisite for translocation of cargo-loaded Pex7p across the peroxisomal membrane (Hensel et al., 2011). In this context, it is also interesting to note that the binding capacity for functional PTS-receptors at the peroxisomal membrane seems to be restricted. The attenuation of receptor export by functional impairment of the exportomer results in an accumulation of *S. cerevisiae* receptors at the membrane (Platta et al., 2004; Kiel et al., 2005) and therefore prevents the docking of incoming receptor-cargo complexes arriving from the cytosol. This assumption is in line with recent data from work in *A. thaliana* (Ratzel et al., 2011). The physiological defects of mutated and only insufficiently active Pex6p were partially restored when combined with a weakly expressed allele of the docking protein Pex13p (Ratzel et al., 2011). This strongly argues for a model in which PTS-receptor import and export rates have to be balanced to allow for a functional matrix protein import cascade.

In summary, the ATP-consuming peroxisomal receptor export machinery is thought to function as import motor for matrix proteins, either indirectly via balanced receptor import/export rates or directly via a linkage of cargo translocation with receptor ubiquitination and export.

## Concluding remarks

The import of peroxisomal matrix proteins differs significantly from other organelles like mitochondria or chloroplasts as peroxisomes can accommodate folded and oligomeric proteins that are targeted to the membrane via cycling receptors.

Much progress has been made in the understanding of the molecular requirements for recognition of the PTS1-signal of peroxisomal matrix proteins by the import receptor Pex5p, for which even several atomic structures are now available. In contrast our knowledge of the structure of Pex7p and mechanism of cargo recognition is still limited. It also still needs to be addressed whether cargo recognition by the peroxisomal import receptors and peroxisomal targeting of the cargo-loaded receptor occurs more or less haphazardly in the cytosol or is also well regulated as indicated by the above mentioned studies on mRNA targeting.

It is now well accepted that the PTS1 receptor Pex5p cycles between a soluble cytosolic state and a membrane-bound state and recent evidence indicated that the receptor at the membrane forms an integral part of the temporally formed import pore. Clearly, it will be of importance to elucidate the assembly of this pore as well as the modulation of its dynamics and gating by regulatory proteins or posttranslational modifications. Apart from this, it will be a significant advance to clarify the existence of an independent PTS2-selective import pore. The functional role of the translocon-associated proteins, e.g., the Pex17p-like and Pex13p-like peroxins or Pex8p is not really understood and needs to be experimentally addressed.

The collected data on the concerted action of sub-complexes of the peroxisomal protein import machinery has merged into the unified model of the receptor export machinery, the exportomer.

In light of its important contribution to the energy requirement of peroxisomal protein import and the proposed export-driven import model, the molecular dynamics of the exportomer during cargo translocation and export of the ubiquitinated receptor need to be investigated. Along this line, one of the most intriguing questions concerns the fact that the PTS1-receptor Pex5p as well as the PTS2-coreceptors Pex18p and Pex20p are ubiquitinated on a cysteine via a thioester bond and not by a more common isopeptide bond to a lysine. The functional relevance of this uncommon kind of ubiquitination remains to be disclosed.

Certainly, many questions regarding the molecular mechanism of matrix protein import into peroxisomes remain to be answered. This is also true for a possible cross-regulation of matrix protein import with other cellular processes, e.g., cellular redox-balance, nutrient and energy status as well as peroxisome maturation, division and degradation.

### Conflict of interest statement

The authors declare that the research was conducted in the absence of any commercial or financial relationships that could be construed as a potential conflict of interest.
